# A Culturally Adapted Smoking Cessation App (IndigeQuit) for American Indian and Alaska Native Adults: Protocol for a Randomized Clinical Trial

**DOI:** 10.2196/102675

**Published:** 2026-09-02

**Authors:** Jonathan B Bricker, Margarita Santiago-Torres, Brianna M Sullivan, Kristin E Mull, Hershel W Clark, Chase Kornacki, Trivia Afraid of Lightning-Craddock, Dean S Seneca, Crystal M Stanford, Sierra L Wilcox, Patricia Nez Henderson, Lonnie Nelson

**Affiliations:** 1 Fred Hutch Cancer Center Seattle, WA United States; 2 Department of Psychiatry and Behavioral Sciences University of Washington Seattle, WA United States; 3 Cancer Consortium, IndigeQuit Community Advisory Board Seattle, WA United States; 4 Navajo Nation Window Rock, AZ United States; 5 Mniconjou Mato Tipila, WY United States; 6 Lakotah Mato Tipila, WY United States; 7 Seneca Nation Salamanca, NY United States; 8 Seneca Scientific Solutions+ Cattaraugus, NY United States; 9 Department of Public Health Sciences University of Rochester Medical Center Rochester, NY United States; 10 School of Public Health and Health Professions University at Buffalo, State University of New York Buffalo, NY United States; 11 Chiricahua Apache Santa Clara, NM United States; 12 Black Hills Center for American Indian Health Rapid City, SD United States; 13 Cherokee Nation Tahlequah, OK United States

**Keywords:** American Indian, Alaska Native, ceremonial tobacco, commercial cigarettes, cultural adaptation, iCanQuit, Indigenous, IndigeQuit, Native, smartphone apps, smoking cessation, tailoring, tribal communities

## Abstract

**Background:**

Due to limited access to evidence-based cessation support, American Indian and Alaska Native (AI/AN) adults are half as likely to quit commercial cigarette smoking as other racial and ethnic groups. Geographical barriers, underfunded health systems, and limited integration of cessation services into routine care have reduced access to effective treatment in AI/AN communities. These challenges are compounded by a lack of culturally relevant interventions tailored to AI/AN adults. Thus, there is an urgent need for accessible, scalable, and culturally relevant interventions.

**Objective:**

Here, we describe the protocol for a randomized clinical trial (RCT) testing the efficacy of a culturally adapted smoking cessation app (IndigeQuit) developed specifically to help AI/AN adults quit smoking commercial cigarettes compared to a standard, nontailored app (QuitGuide).

**Methods:**

To improve the relevance and acceptability of cessation support to AI/AN adults, IndigeQuit was developed through a cultural adaptation of iCanQuit, an evidence-based smartphone app grounded in acceptance and commitment therapy that teaches skills for accepting cravings to smoke. The cultural adaptation used a user-centered, community-based participatory research mixed methods approach in collaboration with a community advisory board (CAB) comprising AI/AN individuals. Cultural adaptations included the use of Native imagery; stories featuring AI/AN adults and elders emphasizing culture, spirituality, family, and community; and the important distinction between ceremonial and commercial tobacco. A total of 776 AI/AN adults who smoke and want to quit are being recruited nationwide and randomized to receive IndigeQuit or QuitGuide for 12 months. The primary aim of the RCT is to determine the efficacy of IndigeQuit compared with QuitGuide for 30-day abstinence at 12 months. Secondary aims include abstinence at earlier time points, identifying mediators and moderators of treatment effects, and assessing engagement and satisfaction. Qualitative interviews with IndigeQuit participants and CAB members will inform the development of a subsequent guide to support the broad dissemination of IndigeQuit nationwide.

**Results:**

The National Cancer Institute funded this study in 2024 (grant R01 CA284687), with the grant awarded to the principal investigator, JBB. As of August 2026, a total of 590 AI/AN adults had been enrolled in the trial. Data collection started in July 2025 and is expected to be completed by November 2028.

**Conclusions:**

The IndigeQuit app was designed to deliver evidence-based smoking cessation treatment that is culturally adapted to AI/AN communities and grounded in acceptance and commitment therapy. If effective, this intervention could offer a more scalable and culturally relevant treatment to AI/AN communities nationwide, helping to reduce smoking-related health inequities.

**Trial Registration:**

ClinicalTrials.gov NCT06145763; https://clinicaltrials.gov/study/NCT06145763

**International Registered Report Identifier (IRRID):**

DERR1-10.2196/102675

## Introduction

### Background

Complex historical factors have contributed to American Indian and Alaska Native (AI/AN) communities having the highest prevalence of commercial cigarette smoking among all racial groups in the United States [1-3]. European colonization, federal policies, and assimilation all disrupted ceremonial tobacco practices, traditionally a sacred ritual in many AI/AN communities, leading to the replacement of ceremonial tobacco with commercial cigarettes [4-11]. Another major driver of these smoking-related inequities is the continued predatory marketing of tobacco products specific to AI/AN communities. Consequently, commercial cigarette smoking is responsible for approximately half of all deaths among AI/AN communities [12-14].

Due to gaps in access to evidence-based cessation support, AI/AN adults are only half as likely to quit commercial cigarette smoking as individuals from other racial and ethnic groups [15]. Geographic barriers to treatment (rural or tribal lands), underfunded health care systems (eg, Indian Health Service), and the lack of integration of cessation services into routine care have contributed to the limited availability of evidence-based treatments in AI/AN communities [16-19]. Compounding these access challenges is the lack of effective, culturally relevant interventions that address the unique needs of AI/AN adults seeking to quit commercial cigarette smoking. Few such programs have been rigorously evaluated [20,21], and the only potentially efficacious randomized clinical trial (RCT) involved an intensive, in-person intervention that may be difficult to disseminate [21]. Therefore, there is an urgent need for accessible, scalable interventions with cultural relevance to this population.

One promising approach to expanding access to culturally relevant, evidence-based cessation treatments among AI/AN communities is through smartphone-based apps [22]. Apps can be freely accessed through an app store and are available anytime at the convenience of the user, representing a scalable, low-barrier delivery modality. Compared to websites and text messaging, smartphone apps have more engaging features, and engagement is a strong predictor of smoking cessation [23-28]. Our group demonstrated the efficacy of the iCanQuit app (based on acceptance and commitment therapy [ACT]) relative to the QuitGuide app (based on the US Clinical Practice Guidelines) for 12-month smoking cessation among 2415 adults from all 50 US states (28.2% vs 21.1% 30-day prevalence quit rate; *P*<.001) [29]. A subsequent secondary analysis among AI/AN adults showed promising results in terms of reach (169 across 32 US states, with 25% residing on tribal lands), 12-month retention (93%), and quit rates (30% vs 18%; *P*=.08). Although quit rates did not differ statistically between the treatments, iCanQuit was significantly more engaging and satisfying, with 80% of iCanQuit vs 56% of QuitGuide AI/AN participants reporting that the app “was made for someone like me” [30].

Given the importance of cultural relevance and acceptability of cessation programs in promoting engagement, and ultimately improving cessation, we culturally adapted iCanQuit, an app-based intervention grounded in ACT, for AI/AN adults (named “IndigeQuit”). The user-centered, community-based participatory research mixed methods approach used to culturally adapt iCanQuit for AI/AN adults has been described in detail elsewhere [31]. Briefly, the adaptation process was informed by a community advisory board (CAB) composed of AI/AN community members and user experience testing among the intended population. Adaptations included refinements to content, imagery, and stories featuring elders emphasizing culture, spirituality, family, and community, as well as incorporating a clear distinction between ceremonial and commercial tobacco use. The core therapeutic components of iCanQuit [29,32] were retained to preserve the efficacy demonstrated in prior studies among the general US population [33]. Accordingly, IndigeQuit continues to target 2 key ACT-based processes: acceptance and values [34-36]. The acceptance component teaches skills for responding to smoking urges through acceptance, whereas the values component helps individuals identify and connect with personally meaningful life domains, such as culture, spirituality, family, and community, thereby strengthening motivation for quitting smoking.

### Objectives

Here, we describe the protocol for an RCT testing the efficacy of IndigeQuit relative to QuitGuide among a planned sample of 776 AI/AN adults recruited nationwide across the United States. The primary cessation aim is to test whether IndigeQuit provides significantly higher quit rates than QuitGuide at 12 months after randomization. Secondary aims include evaluating abstinence at earlier time points and assessing engagement and satisfaction. Qualitative interviews with IndigeQuit participants and CAB members will inform the development of a comprehensive guide to support the broad dissemination of IndigeQuit to AI/AN adults nationwide.

## Methods

### Overview of the IndigeQuit Trial

The IndigeQuit trial is a 12-month, 2-arm, parallel-group RCT with three objectives: (1) to culturally adapt the iCanQuit (Fred Hutchinson Cancer Center) smoking cessation app for AI/AN adults who smoke commercial cigarettes, (2) to determine the efficacy of IndigeQuit relative to the QuitGuide app (National Cancer Institute) for achieving biochemically verified 30-day point prevalence abstinence (PPA) at 12 months, and (3) to develop a comprehensive guide to support the broad dissemination of IndigeQuit among AI/AN adults nationwide.

### Ethical Considerations

Study procedures were approved by the institutional review board at Fred Hutchinson Cancer Center (FHIRB0020279 and RG1123796), and the study was registered at ClinicalTrials.gov (NCT06145763). Informed consent is obtained from all participants, and data are deidentified and stored in a password-protected format. Participants can receive up to US $216 in total incentives over the course of the study, including payments for survey completion, timely survey responses, and saliva test completion (if selected).

Institutional support of trial monitoring will be in accordance with the Fred Hutchinson Cancer Center Institutional Data and Safety Monitoring Plan. The trial will comply with the standard guidelines set forth by these committees and other institutional, state, and federal guidelines. Data and safety monitoring reports will be reviewed monthly by the principal investigator (PI) during the trial. A Data Safety Monitoring Board will not be required because this is a minimal-risk behavioral intervention. Throughout the study, the PI, coinvestigators, and project manager will monitor participants for adverse events and protocol compliance. The project manager will complete monthly reports on participant progress and status, any adverse events, and any protocol deviations.

### Specific Aims

#### Primary Cessation Aim

The primary cessation aim is to determine the efficacy of IndigeQuit relative to QuitGuide for 30-day PPA from commercial cigarette smoking at 12 months after randomization.

#### Secondary Cessation Aim

The secondary cessation aim is to compare 30-day PPA at the 3- and 6-month follow-ups and repeated self-reported 30-day PPA at all time points between treatment arms.

#### Mediation Aim

The mediation aim is to determine whether the effect of IndigeQuit relative to QuitGuide on the 12-month primary cessation outcome is mediated by ACT-based processes of accepting physical sensations, emotions, and thoughts that cue smoking.

#### Moderation Aim

The moderation aim is to explore whether the effect of IndigeQuit relative to QuitGuide on the primary cessation outcome differs by the following baseline factors: (1) sex, (2) income, and (3) smoking intensity.

#### Exploratory Dissemination Aim

The exploratory dissemination aim is to conduct qualitative interviews with a subsample of IndigeQuit participants to thematize testimonials of their experiences with IndigeQuit and AI/AN members of the trial CAB to inform the development of a comprehensive guide to support the broad dissemination of IndigeQuit among AI/AN adults nationwide.

### Participants, Recruitment, and Randomization

#### Eligibility

Eligibility criteria include (1) self-identification as AI/AN, either alone or in combination with other races; (2) age ≥18 years; (3) daily smoking for the past year; (4) interest in quitting smoking; (5) willingness to be randomly assigned to either treatment; (6) daily access to a personal smartphone; (7) ability to download a smartphone app; (8) ability to read English; (9) no current use of or use within the past 30 days of other smoking cessation interventions; (10) no prior participation in our research; and (11) no other household or family member participating in the study.

#### Recruitment and Enrollment

Guided by the CAB, culturally tailored recruitment is conducted primarily via social media advertisements using two main recruitment strategies: (1) using images of AI/AN community members and (2) selecting interests among Facebook (Meta Platforms Inc) and Instagram (Meta Platforms Inc) users associated with an array of interests associated with AI/AN communities and cultures, as recommended by the CAB. Advertisements have also been placed on news sites and at events for AI/AN communities (eg, Native News Online, Indigenous Wellness Institute events, and the Lakota Nation Invitational). Recruitment advertisements are closely monitored and modified as needed based on progress made. We also culturally tailored the recruitment flyer for in-person events and the study website for AI/AN communities. This website provides (1) information about the study; (2) information highlighting that the app was created *by Natives for Natives*; (3) information about the study team, study CAB, Fred Hutchinson Cancer Center, and frequently asked questions (FAQs); and (4) a secure portal to the consent form, screening, baseline assessment, and enrollment into the trial.

Those who are eligible after screening are sent an email inviting them to complete a secure online survey to provide informed consent and complete the baseline assessment. Several precautions are also implemented to deter online fraud: (1) CAPTCHA authentication is used; (2) duplicate or suspicious IP addresses cause ineligibility; and (3) research staff call participants if survey response times, email, or preferred communication method appear suspicious. Those not completing the online enrollment process within 14 days are sent an email notifying them of study ineligibility and providing both the Smokefree.gov website and the 800-QUIT-NOW phone number. Those completing the enrollment process are emailed a secure link with instructions to download their password-protected random assignment: either IndigeQuit or QuitGuide.

#### Randomization and Blinded Allocation

**Figure 1:**
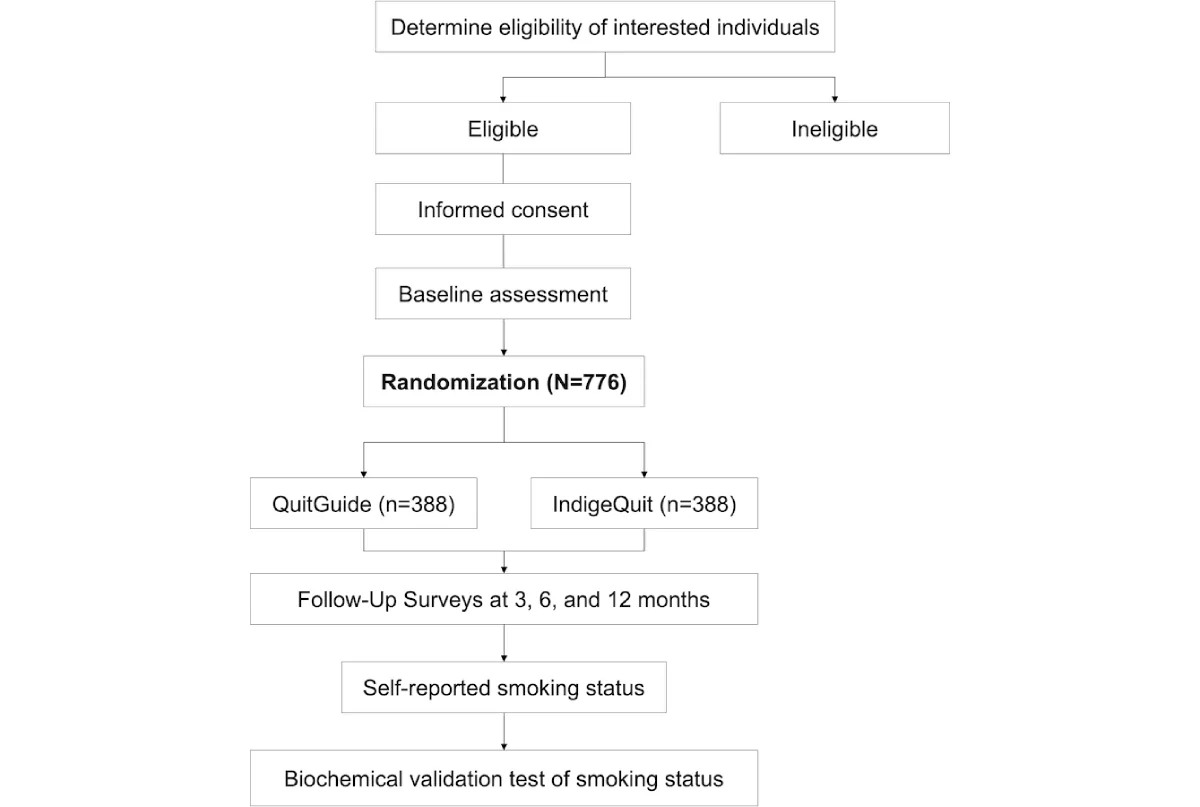
Experimental study design schema.

Eligible individuals are randomized 1:1 to receive access to either the IndigeQuit or QuitGuide app for 12 months (Figure 1). Randomly permuted block randomization is used, with 3 binary stratification factors (ie, sex, income, and smoking intensity) classifying participants into 8 strata. Within each stratum, blocked randomization is implemented using randomly ordered block sizes of 2, 4, and 6 in a 1:3:1 ratio. Random assignments are concealed from participants throughout the trial, and both interventions are branded as “IndigeQuit.” Research staff are blinded to random assignment, and treatment allocation remains concealed until data collection is completed.

### Interventions

#### Active Control Treatment Arm: QuitGuide

As in the original iCanQuit trial [29], the comparison active control app is the National Cancer Institute’s QuitGuide, which follows the US Clinical Practice Guidelines for smoking cessation [37]. On the basis of standard behavioral therapy, QuitGuide helps users develop a quit plan; identify smoking behaviors, triggers, and reasons for being smoke-free; and identify sources of social support for quitting. The app teaches skills for avoiding situations that lead to cravings to smoke, staying smoke-free, and coping with slips and motivates users to quit by providing information on the health consequences of smoking and the benefits of quitting.

Main components of QuitGuide include the following:

- “Thinking about quitting,” which encourages users to think of motivations for quitting and provides information on the general health consequences of smoking
- “Preparing to Quit,” which helps users develop a quit plan; identify smoking behaviors, triggers, and reasons for being smoke-free; identify social support for quitting; and provides information on Food and Drug Administration (FDA)–approved cessation medications
- “Quitting,” which teaches skills for avoiding cravings to smoke, such as finding replacement behaviors and staying busy
- “Staying Quit,” which has tips, motivations, and actions to stay smoke-free and skills for coping with slips via fighting cravings and trying to be positive

Refer to Table 1 and Textbox 1 for a complete comparison between QuitGuide and IndigeQuit.

**Table 1:** Major differences between the QuitGuide and IndigeQuit smartphone apps for smoking cessation.

Major differences	QuitGuide	IndigeQuit
Approach to triggers to smoke	Avoidance: actively trying not to experience urges, emotions, and thoughts that trigger smoking (eg, advice on avoiding triggers and staying busy and suggestions for distracting yourself during an urge)	Acceptance: openness to experience urges, emotions, and thoughts that trigger smoking (eg, experiential exercises and tips for letting urges come and go and progress tracking)
Approach to motivating users to quit	Expectancies: beliefs about what actions will produce goals, information (eg, listing ingredients of a cigarette), risks of secondhand smoke, and rewards (eg, health progress based on smoke-free days)	Values: chosen life directions that guide goals and actions (eg, family and health), testimonials from fictional characters (avatars), and rewards (eg, visual “badges” for smoke-free days)
Approach to relapse prevention	Avoidance: avoid high-risk situations (eg, avoid places where you used to smoke) and avoid urges (eg, advice on how to fight cravings)	Acceptance: perspective-taking (eg, writing a letter from your smoke-free future self) and values (eg, smoke-free vision statement)

**Cultural adaptations and major similarities between QuitGuide and IndigeQuit smartphone apps for smoking cessation.**

**Cultural adaptations *only* in IndigeQuit**

- Culturally relevant content distinguishing ceremonial from commercial tobacco use
- Imagery featuring avatars representative of American Indian and Alaska Native (AI/AN) community members, including the guide avatar Winona (modeled as a community’s older adult), and the Medicine Wheel as the home screen design
- Adapted language reflecting how AI/AN people commonly speak, including “rez” phrasing
- Incorporation of Lakota language phrases to enhance familiarity and credibility of the guide avatar, Winona
- Daily push notifications to promote engagement, interspersed with inspirational quotes from Native leaders and poets that align with the principles of acceptance and commitment therapy
- A chat feature to facilitate user connection and support
- A “Medicines” tool for tracking both traditional and Western medicines
- Emphasis on “honoring the Earth” as a motivational value to support commercial smoking cessation

**Major similarities between QuitGuide and IndigeQuit**

- Education and skills for preparing to quit and preventing relapse, including self-compassion, learning, and starting again
- Intention formation, including setting a quit date as part of an actionable plan for quitting
- Education on Food and Drug Administration–approved smoking cessation medications
- Skills for coping with cravings to smoke
- Education on common triggers to smoke and barriers to cessation, nicotine withdrawal reactions, and how to seek support
- A step-by-step guide with content written at a fifth-grade reading level

#### Experimental Treatment Arm: IndigeQuit

**Figure 2:**
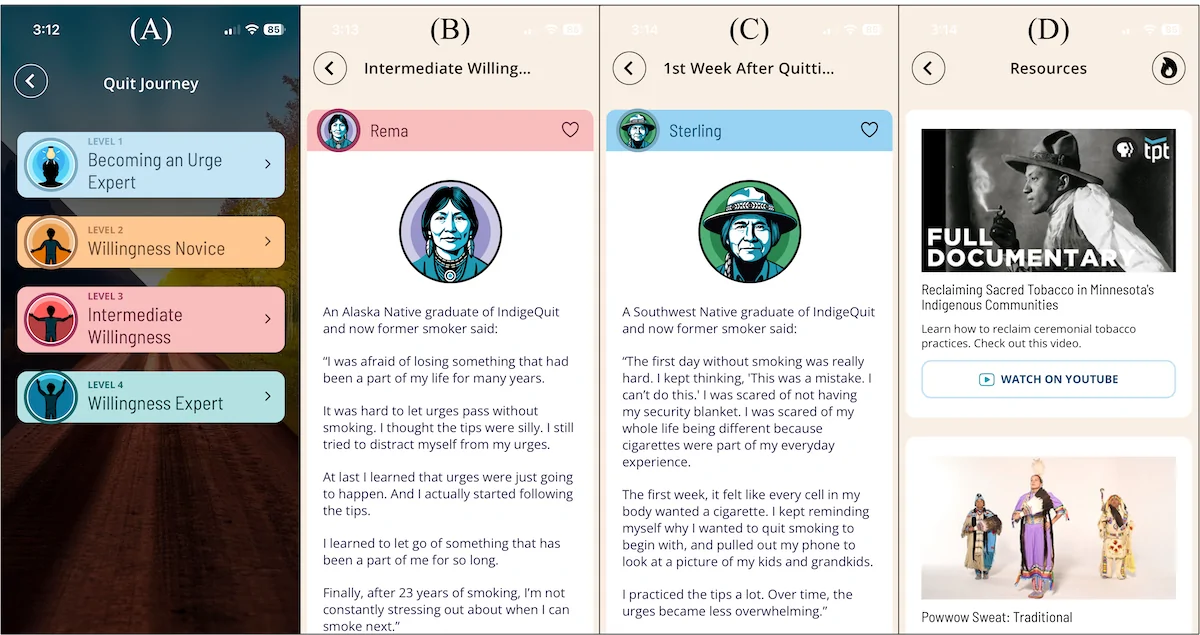
IndigeQuit screenshots, including examples of content culturally tailored to American Indian and Alaska Native adults who smoke. (A) The first 4 levels of content, which focus on preparing to quit and are collectively called “Quit Journey.” Users progress through the 4 levels to develop skills for accepting their urges to smoke. (B) and (C) Examples of avatar testimonials interspersed within the content and exercises. (D) Examples of additional resources available to users, including a link to a documentary about sacred tobacco.

IndigeQuit was developed through a cultural adaptation of iCanQuit, an evidence-based smartphone intervention grounded in ACT that teaches skills for coping with smoking urges, staying motivated, and preventing relapse. Additional details of the iCanQuit app are available in prior publications [29,32]. Core therapeutic components and “active ingredients” of iCanQuit were preserved to maintain efficacy, while surface structure elements—including imagery, language, avatar physical features, and testimonials—were adapted to reflect the cultural values, communication styles, and lived experiences of AI/AN communities (Figure 2).

Methods and details of the cultural adaptation process, which included a user-centered, community-engaged mixed methods approach, have been reported in previous work [31]. Briefly, cultural adaptation included content distinguishing ceremonial from commercial tobacco; imagery featuring avatars representative of AI/AN community members, including the guide avatar *Winona*, who resembles a community elder; and the use of the Medicine Wheel as the basis for the home screen design (Table 1). Slang language throughout was also adapted to reflect how AI/AN people commonly speak today (eg, incorporating “rez” phrasing). A chat feature was included to facilitate user connection and support. Existing daily push notifications to promote engagement were interspersed with inspirational quotes from Native leaders and poets that aligned with ACT principles. An existing medications tool was adapted to include tracking for both traditional (eg, ceremony and prayer) and Western medicines (eg, nicotine replacement therapy and bupropion). Finally, “honoring the Earth” was added to the personal values list as a motivation to support commercial smoking cessation.

### Measures

#### Baseline Assessment

Trial measures and time points are presented in Table 2. Baseline assessment includes sociodemographics, residential location, tribal affiliation, measures of cultural connectedness, discrimination, financial strain, and smoking behaviors, including use of ceremonial tobacco.

**Table 2:** Participant measures and the time points at which they are administered.

Measures	Baseline	3 months	6 months	12 months
Eligibility screening	✓			
Randomization	✓			
Sociodemographics	✓			
Residential location and tribal affiliation	✓			
Cultural connectedness	✓			
Discrimination and financial strain	✓			
Acceptance of cues	✓	✓	✓	
Ceremonial tobacco use	✓	✓	✓	✓
Nicotine, tobacco, e-cigarettes, and cannabis use	✓	✓	✓	✓
Commercial cigarette dependence	✓	✓	✓	✓
Cessation pharmacotherapy use	✓	✓	✓	✓
Quit attempts	✓	✓	✓	✓
Self-reported smoking status	✓	✓	✓	✓
Biochemical validation test of smoking status		✓	✓	✓
Treatment engagement	✓	✓	✓	✓
Treatment satisfaction			✓	✓

#### Smoking Abstinence

##### Primary Cessation Outcome

The primary cessation outcome is 30-day PPA from commercial cigarette smoking at 12 months after randomization. Smoking abstinence is assessed via self-report at the 3-, 6-, and 12-month follow-ups, with biochemical validation in a 10% random subsample using a mailed Alere test to detect cotinine in saliva [38,39]. Participants are asked to take the test and upload photos of themselves taking it and their test results in a secure online survey [40]. Anyone having difficulty with the online survey can contact study staff, who will help participants conduct the test and display their results using a Health Insurance Portability and Accountability Act (HIPAA)–compliant live videoconferencing website. Participants who self-report abstinence but also report using nicotine replacement therapy or e-cigarettes will not be classified as smoking solely based on elevated cotinine, to reduce false positives.

##### Secondary Cessation Outcomes

Secondary outcomes include 30-day PPA from commercial cigarette smoking at the 3- and 6-month follow-ups and repeated 30-day biochemically confirmed PPA across all time points. Additional cessation outcomes include self-reported 24-hour, 7-day, and 30-day PPA and abstinence from other commercial nicotine-containing tobacco products (eg, e-cigarettes or vaping, chewing tobacco, snus, hookahs, cigars, cigarillos, tobacco pipes, and *kreteks*) at the 3-, 6-, and 12-month follow-ups.

#### Potential Mediators

Acceptance has been identified as a core mediator of treatment effect in prior smoking cessation interventions [30,41]. Acceptance of internal cues to smoke is measured via the validated Avoidance and Inflexibility Scale [42], using the mean of the three 9-item subscales that assess participants’ willingness to experience physical sensations, emotions, and thoughts that cue smoking. The items are rated on a 5-point Likert scale (ranging from 1=“Not at all” to 5=“Very willing”) and averaged, with higher scores indicating greater acceptance. A sample sensation item is “How willing are you to notice these sensations without smoking?” The items from the emotions and thoughts subscales are similar, substituting “feelings” or “thoughts” for “sensations.”

#### Potential Moderators

Potential moderators of the primary cessation outcome are assessed at baseline and include (1) sex (male vs female), (2) annual income (US <$20,000 vs US ≥$20,000), and (3) smoking intensity (≤10 vs >10 cigarettes per day).

#### Treatment Engagement and Satisfaction

Treatment engagement with each intervention is measured over the 12-month trial period. The primary engagement measure is the number of times users interact with their assigned intervention (ie, number of logins). Secondary measures of engagement are (1) the number of days from first to last use, (2) the number of unique days of use, and (3) time spent using the app. Satisfaction with the assigned apps is assessed via the 3-month follow-up study questionnaire. Example items include (1) “How useful was your assigned app for quitting smoking?” and (2) “How satisfied were you with your assigned app?”; both items are rated on a 5-point scale (ranging from “not at all” to “very much”).

#### Dissemination

To inform the development of a dissemination guide, we will conduct two sets of semistructured qualitative interviews: (1) with participants in the IndigeQuit arm who have completed the trial and (2) with AI/AN CAB members. Interviews will be conducted via secure videoconference, will last approximately 75 minutes, will be audio-recorded, and will be professionally transcribed verbatim. Participants will receive US $75 for completing the interview.

For IndigeQuit participants, we will recruit a 10% random sample (n=39) stratified by app use (no logins vs ≥1 login) and smoking status at 12 months (abstinent vs not abstinent). Interviews will follow a standardized semistructured interview guide developed collaboratively with the study CAB and a multidisciplinary team of AI/AN and non-AI/AN investigators with expertise in qualitative research, behavioral interventions, digital health, and commercial tobacco cessation. Trial data on recruitment yield, participant engagement, and satisfaction with IndigeQuit will inform interview guide development while allowing flexibility to explore emergent topics in greater depth. Example topics include (1) preferred ways to seek help for smoking cessation, (2) experiences using IndigeQuit, (3) perceptions of what makes the intervention unique, and (4) barriers to app use among nonusers.

For interviews with AI/AN CAB members representing diverse geographic regions, tribal affiliations, and professional roles (eg, Indian Health Service and tobacco control specialists), questions will focus on (1) barriers and facilitators to dissemination; (2) strategies for national dissemination to AI/AN communities; and (3) how high-performing recruitment advertisements, identified through trial data on reach and cost per randomization, could be adapted for large-scale dissemination. For example, CAB members will be asked whether messaging, imagery, cultural references, testimonials, or dissemination channels should be modified to increase relevance and engagement across diverse AI/AN communities and settings while preserving elements associated with strong recruitment performance.

Interview transcripts will be analyzed using the Braun and Clarke [43,44] reflexive thematic analysis approach [45]. Coding and theme development will be iterative and inductive, with regular discussions among the research team to promote flexibility and transparency in interpretation. Researchers will consider how their backgrounds, perspectives, and relationships with the research may influence data collection, coding, and interpretation.

Qualitative and quantitative findings will be integrated using a mixed methods approach. Quantitative data will characterize recruitment reach and efficiency, intervention engagement, satisfaction, and smoking outcomes. Qualitative data will help explain these patterns and identify perceived dissemination barriers, facilitators, and preferences. Integrated findings will be used to refine and prioritize recommendations and inform the development of the dissemination guide.

### Statistical Analysis Plan

#### Sample Size

The study sample size of 776 AI/AN adults was calculated based on conservative estimates of outcome data retention and quit rates from our secondary analysis of data from this subgroup in the iCanQuit trial [30]. As is common in smoking cessation trials, all participants with missing outcome data will be considered nonabstinent [46,47]. The study design includes 1:1 randomization of participants to treatment arms, 2-sided tests with α=.05, and estimated 12-month 30-day PPA rates of 17.1% and 10.2% in the IndigeQuit and QuitGuide arms, respectively. Under these assumptions, 80% power is achieved with 388 participants randomized to each arm.

#### Approach

Participant sociodemographic, mental health, smoking behavior, and cultural factors at baseline will be summarized for each treatment arm. Logistic regression models will be used to estimate the treatment effect for each binary smoking cessation outcome, adjusting for factors used in stratified randomization and baseline factors significantly related to the outcome [48,49]. Planned sensitivity analyses include complete-case cessation outcomes at each follow-up time point and multiple imputation [50] of missing primary outcome data at 12 months. Adjusted negative binomial regression models will be used to assess the difference between treatment arms for right-skewed count outcomes, such as the number of app sessions. Moderation of the treatment effect by sex, income, and smoking intensity will be assessed through separate adjusted logistic regression models with treatment-by-moderator interaction terms. Significant interactions will be probed with subgroup analyses to estimate the treatment effect at each value of the moderator. Finally, to test whether the effect of IndigeQuit on the primary smoking cessation outcome is mediated by changes in acceptance of cravings to smoke, we will use the PROCESS macro with bootstrap sampling to estimate the mediation effect and its 95% CI [51,52]. All statistical tests will be 2-sided with α=.05.

## Results

The trial was funded by the National Cancer Institute in August 2024. As of August 2026, a total of 590 AI/AN adults who smoke commercial cigarettes had been enrolled. Data collection started in July 2025 and is expected to be completed by November 2028. Results will be disseminated through peer-reviewed publications and presentations at national and international scientific conferences.

## Discussion

### Anticipated Findings

The IndigeQuit RCT was designed to culturally adapt an effective smoking cessation app (iCanQuit) for AI/AN adults and evaluate the efficacy of IndigeQuit relative to a nontailored app (QuitGuide) among 776 AI/AN adults recruited nationwide. This project will deliver the first definitive trial of a promising digital intervention with the potential to reach AI/AN communities nationwide—a population that has long experienced disproportionate burdens of commercial cigarette smoking and smoking-related cancer mortality [13,53]. The study will advance scientific understanding of the efficacy of a culturally adapted app to support commercial smoking cessation among AI/AN individuals and evaluate its scalability for broad dissemination. If effective, this intervention could meaningfully advance health equity by providing an accessible, evidence-based tool to support commercial smoking cessation among AI/AN adults across diverse geographic settings.

### Strengths

The strengths of this trial include its rigorous randomized clinical study design with double-blind allocation and an active control condition, a fully powered sample size to detect commercial cigarette smoking cessation outcomes, and long-term follow-up to assess sustained effects. The study is further strengthened by biochemical verification of smoking status and the use of validated assessment measures. Its community-engaged approach, including community-based participatory research and an active CAB, together with community-informed cultural adaptation of the intervention, ensures relevance and acceptability. Additional strengths include nationwide recruitment, which supports generalizability, the examination of treatment mediators and moderators, and an exploratory dissemination aim to inform future uptake of IndigeQuit nationwide.

### Challenges and Potential Limitations

Potential challenges include trial recruitment, app engagement, retention, and remote biochemical verification. To support recruitment, we are implementing social media strategies proven effective in prior nationwide digital cessation trials, with ongoing CAB guidance to refine outreach and ensure cultural relevance. To enhance engagement and retention, we have implemented a multimodal survey procedure and continue to collect collateral contact information and provide tiered incentives, including preintervention incentives and 24-hour completion bonuses. Importantly, these approaches achieved a 93% follow-up rate among AI/AN participants in the iCanQuit parent trial [30,54,55]. CAB members will remain actively involved throughout to help address emerging recruitment and retention challenges. To address remote biochemical verification challenges, we applied prior qualitative research insights on key barriers and implemented a participant-centered approach to enhance engagement and reciprocity, alongside staff-supported procedures to improve troubleshooting, sample return rates, and reporting accuracy [56].

### Conclusions

The IndigeQuit app was designed to deliver evidence-based smoking cessation treatment that is culturally adapted to AI/AN communities and grounded in ACT. If effective, this intervention could offer a more scalable and culturally relevant treatment to AI/AN communities nationwide, helping to reduce smoking-related health inequities. In addition, qualitative interviews will identify key barriers and facilitators to dissemination, informing the development of a comprehensive guide to support the broad dissemination of IndigeQuit.

## Data Availability

The data will be shared upon reasonable request to JBB.

## Appendix

Multimedia Appendix 1 Peer-Review Report by HPC - Health Promotion in Communities Study Section, Healthcare Delivery and Methodologies Integrated Review Group, National Institute of Cancer (National Institutes of Health, USA).

## Abbreviations

ACT: acceptance and commitment therapy
AI/AN: American Indian and Alaska Native
CAB: community advisory board
FAQ: frequently asked question
FDA: Food and Drug Administration
HIPAA: Health Insurance Portability and Accountability Act
PI: principal investigator
PPA: point prevalence abstinence
RCT: randomized clinical trial

## References

[1] (2024). American Indian and Alaska native people and commercial tobacco: health disparities and ways to advance health equity. Centers for Disease Control and Prevention.

[2] United States Public Health Service Office of the Surgeon General, National Center for Chronic Disease Prevention and Health Promotion (US) Office on Smoking and Health (2020). Smoking Cessation: A Report of the Surgeon General.

[3] (2024). Current cigarette smoking among adults in the United States. Centers for Disease Control and Prevention.

[4] Nez Henderson P, Lee JP, Soto C, O Leary R, Rutan E, D Silva J, Waa A, Henderson ZP, Nez SS, Maddox R (2022). Decolonization of tobacco in indigenous communities of Turtle Island (North America). Nicotine Tob Res.

[5] Maddox R, Waa A, Lee K, Nez Henderson P, Blais G, Reading J, Lovett R (2019). Commercial tobacco and indigenous peoples: a stock take on Framework Convention on Tobacco Control progress. Tob Control.

[6] Maddox R, Bovill M, Waa A, Gifford H, Tautolo ES, Nez Henderson P, Martinez S, Clark H, Bradbrook S, Calma T (2022). Reflections on indigenous commercial tobacco control: 'the dolphins will always take us home'. Tob Control.

[7] Margalit R, Watanabe-Galloway S, Kennedy F, Lacy N, Red Shirt K, Vinson L, Kills Small J (2013). Lakota elders' views on traditional versus commercial/addictive tobacco use; oral history depicting a fundamental distinction. J Community Health.

[8] Kunitz SJ (2016). Historical influences on contemporary tobacco use by Northern Plains and Southwestern American Indians. Am J Public Health.

[9] Wilson J, Sabo S, Chief C, Clark H, Yazzie A, Nahee J, Leischow S, Nez Henderson P (2019). Diné (Navajo) healer perspectives on commercial tobacco use in ceremonial settings: an oral story project to promote smoke-free life. Am Indian Alsk Native Ment Health Res.

[10] D'Silva J, O'Gara E, Villaluz NT (2018). Tobacco industry misappropriation of American Indian culture and traditional tobacco. Tob Control.

[11] Lempert LK, Glantz SA (2019). Tobacco industry promotional strategies targeting American Indians/Alaska Natives and exploiting tribal sovereignty. Nicotine Tob Res.

[12] Espey DK, Jim MA, Cobb N, Bartholomew M, Becker T, Haverkamp D, Plescia M (2014). Leading causes of death and all-cause mortality in American Indians and Alaska Natives. Am J Public Health.

[13] Mowery PD, Dube SR, Thorne SL, Garrett BE, Homa DM, Nez Henderson P (2015). Disparities in smoking-related mortality among American Indians/Alaska Natives. Am J Prev Med.

[14] Odani S, Armour BS, Graffunder CM, Garrett BE, Agaku IT (2017). Prevalence and disparities in tobacco product use among American Indians/Alaska Natives - United States, 2010-2015. MMWR Morb Mortal Wkly Rep.

[15] Babb S, Malarcher A, Schauer G, Asman K, Jamal A (2017). Quitting smoking among adults - United States, 2000-2015. MMWR Morb Mortal Wkly Rep.

[16] Warne D, Frizzell LB (2014). American Indian health policy: historical trends and contemporary issues. Am J Public Health.

[17] Frerichs L, Bell R, Lich KH, Reuland D, Warne DK (2022). Health insurance coverage among American Indians and Alaska Natives in the context of the Affordable Care Act. Ethn Health.

[18] Willging CE, Jaramillo ET, Haozous E, Sommerfeld DH, Verney SP (2021). Macro- and meso-level contextual influences on health care inequities among American Indian elders. BMC Public Health.

[19] Jaramillo ET, Haozous EA, Willging CE (2022). Experiences of health insurance among American Indian elders and their health care providers. J Health Polit Policy Law.

[20] Carroll DM, Jennings D, Stately A, Kamath A, Tessier KM, Cotoc C, Egbert A, Begnaud A, Businelle M, Hatsukami D, Pickner W (2025). Pilot randomised controlled trial of a culturally aligned smoking cessation app for American Indian persons. Tob Control.

[21] Choi WS, Beebe LA, Nazir N, Kaur B, Hopkins M, Talawyma M, Shireman TI, Yeh HW, Greiner KA, Daley CM (2016). All nations breath of life: a randomized trial of smoking cessation for American Indians. Am J Prev Med.

[22] Abroms LC, Lee Westmaas J, Bontemps-Jones J, Ramani R, Mellerson J (2013). A content analysis of popular smartphone apps for smoking cessation. Am J Prev Med.

[23] Harrison R, Flood D, Duce D (2013). Usability of mobile applications: literature review and rationale for a new usability model. J Interact Sci.

[24] Berg CJ (2011). Internet-based interventions for smoking cessation show inconsistent effects across trials, with only some trials showing a benefit. Evid Based Nurs.

[25] Hutton HE, Wilson LM, Apelberg BJ, Tang EA, Odelola O, Bass EB, Chander G (2011). A systematic review of randomized controlled trials: web-based interventions for smoking cessation among adolescents, college students, and adults. Nicotine Tob Res.

[26] Shahab L, McEwen A (2009). Online support for smoking cessation: a systematic review of the literature. Addiction.

[27] Webb TL (2009). Commentary on Shahab and McEwen (2009): understanding and preventing attrition in online smoking cessation interventions: a self-regulatory perspective. Addiction.

[28] Whittaker R, McRobbie H, Bullen C, Borland R, Rodgers A, Gu Y (2012). Mobile phone-based interventions for smoking cessation. Cochrane Database Syst Rev.

[29] Bricker JB, Watson NL, Mull KE, Sullivan BM, Heffner JL (2020). Efficacy of smartphone applications for smoking cessation: a randomized clinical trial. JAMA Intern Med.

[30] Santiago-Torres M, Mull KE, Sullivan BM, Kwon DM, Nez Henderson P, Nelson LA, Patten CA, Bricker JB (2022). Efficacy and utilization of smartphone applications for smoking cessation among American Indians and Alaska Natives: results from the iCanQuit trial. Nicotine Tob Res.

[31] Bricker JB, Santiago-Torres M, Sullivan BM, Mull KE, Clark HW, Fogel CA, Hwang SB, Keith AR, Kornacki C, Afraid Of Lightning-Craddock T, Martinez SA, Seneca DS, Stanford CM, Terry C, Wilcox SL, Nelson L, Henderson PN (2026). Development of a culturally adapted smartphone app (IndigeQuit) designed to help American Indian and Alaska Native people quit commercial cigarettes: user-centered mixed methods study. JMIR Form Res.

[32] Bricker JB, Mull KE, Kientz JA, Vilardaga R, Mercer LD, Akioka KJ, Heffner JL (2014). Randomized, controlled pilot trial of a smartphone app for smoking cessation using acceptance and commitment therapy. Drug Alcohol Depend.

[33] Bricker JB, Mull KE, Sullivan BM, Forman EM (2021). Efficacy of telehealth acceptance and commitment therapy for weight loss: a pilot randomized clinical trial. Transl Behav Med.

[34] Hayes SC (2005). Get Out of Your Mind and Into Your Life: The New Acceptance and Commitment Therapy.

[35] Hayes SC, Luoma JB, Bond FW, Masuda A, Lillis J (2006). Acceptance and commitment therapy: model, processes and outcomes. Behav Res Ther.

[36] Hayes SC, Strosahl KD, Wilson KG, Sandoz EK, Craske MG (2016). Acceptance and Commitment Therapy: The Process and Practice of Mindful Change.

[37] Fiore MC, Jaén CR, Baker TB, Bailey WC, Benowitz NL, Curry SJ (2008). Treating Tobacco Use and Dependence: 2008 Update.

[38] Raja M, Garg A, Yadav P, Jha K, Handa S (2016). Diagnostic methods for detection of cotinine level in tobacco users: a review. J Clin Diagn Res.

[39] Cooke F, Bullen C, Whittaker R, McRobbie H, Chen MH, Walker N (2008). Diagnostic accuracy of NicAlert cotinine test strips in saliva for verifying smoking status. Nicotine Tob Res.

[40] Heffner JL, McClure JB (2022). Commentary on Graham et al.: biochemical verification of abstinence in remotely conducted smoking cessation trials should not be a universal design requirement for rigor. Addiction.

[41] Bricker JB, Levin M, Lappalainen R, Mull K, Sullivan B, Santiago-Torres M (2021). Mechanisms of smartphone apps for cigarette smoking cessation: results of a serial mediation model from the iCanQuit randomized trial. JMIR Mhealth Uhealth.

[42] Gifford EV, Kohlenberg BS, Hayes SC, Antonuccio DO, Piasecki MM, Rasmussen-Hall ML, Palm KM (2004). Acceptance-based treatment for smoking cessation. Behav Ther.

[43] Braun V, Clarke V (2022). Toward good practice in thematic analysis: avoiding common problems and be(com)ing a knowing researcher. Int J Transgend Health.

[44] Braun V, Clarke V (2008). Using thematic analysis in psychology. Qual Res Psychol.

[45] Terry G, Hayfield N, Clarke V, Braun V, Willig C, Rogers WS (2017). Thematic analysis. The SAGE Handbook of Qualitative Research in Psychology.

[46] Whittaker R, McRobbie H, Bullen C, Rodgers A, Gu Y, Dobson R (2019). Mobile phone text messaging and app-based interventions for smoking cessation. Cochrane Database Syst Rev.

[47] Graham AL, Carpenter KM, Cha S, Cole S, Jacobs MA, Raskob M, Cole-Lewis H (2016). Systematic review and meta-analysis of internet interventions for smoking cessation among adults. Subst Abuse Rehabil.

[48] Kernan WN, Viscoli CM, Makuch RW, Brass LM, Horwitz RI (1999). Stratified randomization for clinical trials. J Clin Epidemiol.

[49] Pocock SJ, Assmann SE, Enos LE, Kasten LE (2002). Subgroup analysis, covariate adjustment and baseline comparisons in clinical trial reporting: current practice and problems. Stat Med.

[50] Donald BR (1987). Multiple Imputation for Nonresponse in Surveys.

[51] Hayes AF (2017). Introduction to Mediation, Moderation, and Conditional Process Analysis: A Regression-Based Approach.

[52] Preacher KJ, Hayes AF (2008). Asymptotic and resampling strategies for assessing and comparing indirect effects in multiple mediator models. Behav Res Methods.

[53] Plescia M, Henley SJ, Pate A, Underwood JM, Rhodes K (2014). Lung cancer deaths among American Indians and Alaska Natives, 1990-2009. Am J Public Health.

[54] Bricker JB, Watson NL, Heffner JL, Sullivan B, Mull K, Kwon D, Westmaas JL, Ostroff J (2020). A smartphone app designed to help cancer patients stop smoking: results from a pilot randomized trial on feasibility, acceptability, and effectiveness. JMIR Form Res.

[55] Watson NL, Mull KE, Heffner JL, McClure JB, Bricker JB (2018). Participant recruitment and retention in remote eHealth intervention trials: methods and lessons learned from a large randomized controlled trial of two web-based smoking interventions. J Med Internet Res.

[56] Santiago-Torres M, Mull KE, Sullivan BM, Fogel CA, Hwang SB, Keith AR, David SP, Bricker JB (2026). Overcoming challenges to remote biochemical verification of smoking status: insights from participant interviews. Subst Use.

