# Supplementary material for: A Culturally Adapted Smoking Cessation App (IndigeQuit) for American Indian and Alaska Native Adults: Protocol for a Randomized Clinical Trial
Source: JMIR Res Protoc. 2026 Sep 2;15:e102675. doi: 10.2196/102675 (PMC13583490; doi:10.2196/102675)
Supplement: Supplementary file 1 [file resprot_v15i1e102675_app1.pdf]

SUMMARY STATEMENT
(Privileged Communication)

PROGRAM CONTACT:
JOSEPH Ciccolo
240 276 7052
joseph.ciccolo@nih.gov

Release Date: **07/21/2023**
Revised Date:

Application Number: **1R01CA284687-01A1**
Formerly: **1R01CA284687-01**

Principal Investigator

BRICKER, JONATHAN B

Applicant Organization: FRED HUTCHINSON CANCER CENTER

Review Group: **HPC**
Health Promotion in Communities Study Section

Meeting Date: **06/20/2023**
Council: **OCT 2023**
Requested Start: **12/01/2023**

Opportunity Number: **PAR-21-035**
PCC: **DFTC**

Project Title: **Digital smoking cessation intervention for nationally-recruited American Indians and Alaska Natives: A full-scale randomized controlled trial**
SRG Action: **Impact Score:13 Percentile:1**
Next Steps: **Visit https://grants.nih.gov/grants/next_steps.htm**
Human Subjects: 30-Human subjects involved - Certified, no SRG concerns
Animal Subjects: 10-No live vertebrate animals involved for competing appl.
Gender: 1A-Both genders, scientifically acceptable
Minority: 2A-Only minorities, scientifically acceptable
Age: 3A-No children included, scientifically acceptable

[redacted] [redacted] [redacted]

ADMINISTRATIVE BUDGET NOTE: The budget shown is the requested budget and has not been adjusted to reflect any recommendations made by reviewers. If an award is planned, the costs will be calculated by Institute grants management staff based on the recommendations outlined below in the COMMITTEE BUDGET RECOMMENDATIONS section.

1R01CA284687-01A1 BRICKER, JONATHAN

RESUME AND SUMMARY OF DISCUSSION: This highly responsive resubmitted application proposes to determine the efficacy of a smartphone application aimed at helping American Indians and Alaska Natives (AIANs) quit smoking. Reviewers found the application highly significant in its focus on AIANs, a vulnerable yet understudied population that lacks disseminable smoking cessation interventions. If successful, the intervention could potentially be scaled to reach the large proportion of AIANs with smartphones. Reviewers agreed that this is a stellar investigative team with well aligned expertise. The team's embeddedness in the target community was noted as an additional strength. The research environment is excellent and has the necessary resources to support successful completion of the project's aims. Reviewers noted several strengths with the approach, including a rigorous design that allows examination of culturally and theoretically informed mediators and moderators, strong preliminary data, a strong community advisory board to inform all aspects of the project, and measures and analysis plan that align well with study aims. This was an impressive application from an exceptional group of investigators and enthusiasm was extraordinarily high among reviewers. Overall, findings are likely to have a high impact on advancing smoking cessation interventions among AIANs.

DESCRIPTION (provided by applicant): Since the early 1980s, American Indians and Alaska Natives (AIANs) have maintained the highest rates of commercial cigarette smoking of any racial/ethnic group in the US. Currently, 27% of AIANs smoke cigarettes. Compared to other racial/ethnic groups, they have 6 times higher rates of developing smoking-related cancers. AIANs are only half as likely to quit smoking compared to other races/ethnicities. The result is that commercial cigarette smoking accounts for half of all deaths among AIANs nationwide. A major cause of these longstanding inequities is the lack of access to efficacious smoking cessation interventions among AIANs. Compounding the barrier of lack of access to smoking cessation interventions is the barrier of lack of research on the efficacy of smoking cessation interventions for AIANs. Despite having the highest smoking prevalence of any racial/ethnic group in the US for over 40 years, a mere 0.3% of all full-scale smoking cessation randomized controlled trials (RCTs) have focused on AIANs. Regarding accessibility, a smartphone application ("app") has the potential to deliver a low-cost smoking cessation intervention with wide geographic reach to AIANs and in regions of the US with smoking rates as high as 57% in this group (i.e., Northern Plains). Regarding efficacy, our preliminary data provides promising evidence for our Acceptance and Commitment Therapy (ACT)-based smartphone app, called iCanQuit, to help AIANs quit smoking. We compared the iCanQuit app with the NCI's QuitGuide app among the AIAN subsample (N = 165 recruited from 32 US states) enrolled in our full-scale RCT. This secondary analysis of AIANs showed descriptively higher rates of smoking cessation at the 12-month follow-up (30% for iCanQuit vs. 18% for QuitGuide; OR = 1.96; 95% CI = 0.90, 4.26, $p = .089$). While encouraging, analyses were exploratory, non-significant, and not a substitute for a full-scale efficacy test. To address weaknesses of prior research, a fully-powered comparative efficacy RCT of iCanQuit vs. QuitGuide focusing nationwide on AIANs who smoke is now needed. Thus, the goal of this project is to conduct a nationally recruited and fully-powered two-arm RCT comparing iCanQuit (n = 388) to QuitGuide (n = 388), in order to determine: (1) the efficacy of iCanQuit relative to the QuitGuide app for biochemically verified 30-day point prevalence abstinence (PPA) at 12 months post-randomization and (2) whether iCanQuit's (but not QuitGuide's) 12-month smoking cessation outcomes are significantly mediated by improvements in core ACT-based processes. This study will be the first full-scale RCT of a digital intervention for helping AIANs nationwide stop smoking. Qualitative interviews with (1) a subsample of iCanQuit participants to thematize testimonials of their experience with iCanQuit and (2) AIAN members from our study Community Advisory Board (CAB) will guide our plan for broadly disseminating iCanQuit to AIAN adults nationwide. Positive results would improve health equity by providing a highly accessible and efficacious intervention with potential for sustainability and broad dissemination for AIANs nationwide.

PUBLIC HEALTH RELEVANCE: The overall aim of this project is to determine the efficacy of a smartphone application aimed at helping American Indians and Alaska Natives (AIANs) quit smoking. Positive results would improve health equity by providing a highly accessible and efficacious intervention for AIANs nationwide.

CRITIQUE 1

Significance: 1
Investigator(s): 1
Innovation: 2
Approach: 1
Environment: 1

Overall Impact: This resubmission application builds on the research team's prior iCanQuit trial results. Secondary analysis of this trial testing an Acceptance and Commitment Therapy [ACT]-based smartphone app vs. NCI's QuitGuide app showed that AIANs (n=165) demonstrated high 12-month retention, higher engagement, and marginally higher rates of smoking cessation at 12-month follow-up. The proposed fully powered comparative efficacy RCT of iCanQuit vs. QuitGuide focusing on AIANs (n=298/condition) will determine efficacy (biochemically verified 30-day ppa at 12 months) and mediation. Additionally, qualitative interviews of iCanQuit participants and CAB members will determine potential facilitators and barriers to disseminating iCanQuit. This is a stellar team in well-suited environments. Further, the resubmission application is responsive to the prior set of reviews, by revising aspects of the research plan (e.g., response rates, sample size, recruitment strategy), giving additional attention to dissemination, and further justifying the intervention and comparison conditions. In addition, the resubmission application very clearly addresses the unique needs of and rationale for an intervention study for this population – apart from other populations that showed similar results in secondary analyses of the former trial. In short, this proposal was previously reviewed as generally rigorous in design and based on rigorous prior research, and the rationale, significance, and innovation of this proposal has been clarified and strengthened.

1. Significance:

Strengths

- This resubmission application speaks directly and convincingly to the question previously raised regarding the rationale for the study. The rationale is stated very strongly in terms of why this subpopulation – out of subpopulations addressed in secondary analyses of the prior intervention study – warrants specific follow up, due to this subpopulations' exceptionally high smoking rates and related morbidity and mortality, as well as the particularly lacking rigorous intervention trials to date. It is important to not only address why this subpopulation warrants attention, but also clearly state why it is unique among all of the subpopulations that were studied and showed similar results in the former trial. The research team provided a more compelling argument here.
- Additionally, the expanded rationale for the intervention and clarity regarding modifications made to the intervention has addressed prior concerns related to related comparison decisions.

Weaknesses

- None noted by reviewer.

2. Investigator(s):

Strengths

- This team, led by PI Bricker, is stellar, with an outstanding track record of directly relevant research, complementary areas of strength, and a collaborative history.

Weaknesses

- None noted by reviewer.

3. Innovation:

Strengths

- This study includes several innovations, including the use of a smartphone app-based intervention using ACT for this population, examination of its dissemination, and examination of population-specific mediators and moderators.

Weaknesses

- None noted by reviewer.

4. Approach:

Strengths

- The study team proposes a rigorously-designed study extending findings from this team's prior research.
- The recruitment procedures, sampling, measures, and analyses are well-articulated and sufficiently specific. The measures and analytic plan align with the aims of the study.
- The expanded rationale and increased clarity regarding modifications of the intervention.
- Sex as a biological variable is addressed.

Weaknesses

- None noted by reviewer.

5. Environment:

Strengths

- Well-suited environments to support the proposed research.

Weaknesses

- None noted by reviewer.

Study Timeline:

Strengths

- Appropriate timeline.

Weaknesses

- No significant weaknesses noted.

Protections for Human Subjects:
Acceptable Risks and/or Adequate Protections.

- Appropriately considered.

Data and Safety Monitoring Plan (Applicable for Clinical Trials Only):
Acceptable.

- Appropriately considered.

Inclusion Plans:

- Sex/Gender: Distribution justified scientifically.
- Race/Ethnicity: Distribution justified scientifically.
- For NIH-Defined Phase III trials, Plans for valid design and analysis: Scientifically acceptable.
- Inclusion/Exclusion Based on Age: Distribution justified scientifically.
- Appropriately considered.

Vertebrate Animals:
Not Applicable (No Vertebrate Animals)

Biohazards:
Not Applicable (No Biohazards)

Resubmission:

- Responsive to the prior set of reviews.

Resource Sharing Plans:
Acceptable

- Appropriately considered.

Budget and Period of Support:
Recommend as Requested.

CRITIQUE 2

Significance: 1
Investigator(s): 1
Innovation: 2
Approach: 2
Environment: 1

Overall Impact: This resubmission is very strong on all criteria and appropriately responsive to the prior review. Overall, the major scientific gap, thorough cultural considerations, scientific rigor, practical feasibility, and dissemination planning make the project likely to have high impact. This outweighs the fact that the methods are best-practice but not particularly innovative. The only minor weakness with the approach is that updating the app in 9 months seems ambitious, but this is mitigated by the fact that the PI has worked successfully with the developers/programmers on similar projects previously.

1. Significance:

Strengths

- Very few disseminable cessation interventions for understudied AIANs, despite major tobacco disparities.
- Intervention based on ACT theory, with promising prelim data among AIANs.
- Study will assess theoretical mediators and moderators and begin dissemination planning.
- Rigor of prior research is described.

Weaknesses

- None noted by this reviewer.

2. Investigator(s):

Strengths

- Well-qualified, strong PI.
- History of collaboration with Drs. Nelson (cultural adaptation, Cherokee descendant) and Nez Henderson (MD and Navajo member), both experts in AIAN health disparities and tobacco cessation.
- Santiago-Torres has experience in community engagement.
- Ms. Mull is a statistician with 10 years of experience collaborating with the PI.
- Consultant Clinton Isham is a Tribal Relations Independent Consultant with experience related to tobacco.
- Various members of the team have collaborated previously and have strong community AIAN connections.

Weaknesses

- None noted by this reviewer.

3. Innovation:

Strengths

- First full-scale RCT of a smartphone app for helping AIANs stop smoking.
- App may foster accessibility, and dissemination planning will occur.
- Assessing unique and culturally- and theoretically- informed mediators.

Weaknesses

- Methods are best practice but not particularly innovative.

4. Approach:

Strengths

- Rigor: Multiple strategies for scientific rigor are described, including fraud detection for digital recruitment and participation. Biochemically verified primary outcome at 12 months.
- Cultural relevance and reach: Assessing unique and culturally- and theoretically- informed mediators. Methods of adaptation are appropriate. CAB will contribute to all aspects of the project including planning for dissemination. Apps don't require internet to use, facilitating rural and low SES reach.
- Feasibility: Team has demonstrated successful recruitment of a reasonably large sample of AIANs for iCanQuit pilot. Facebook ads will be targeted to AIANs with quotas to obtain a sufficient number of men. This will be supplemented by connections with Tribal Health Centers and ResearchMatch.com if needed.
- Potential Impact: Prelim data informs and supports adaptation and use of iCanQuit for AIANs. Assessment of reach, recruitment cost, and engagement can inform future D&I.
- Design: Comparing to the NCI QuitGuide is well-justified, and similarities and differences are outlined. Prelim data suggests AIANs may do better with iCanQuit.

Weaknesses

- Updating the app in 9 months seems ambitious, particularly with limited info about Moby (the developers/programmers) and no LOS from them.
- Minor: Not sure if assuming missing data as smoking is state-of-the-science. Aren't there potentially a lot of AIANs who might rather use a web app instead of downloading an app onto their phones?

5. Environment:

Strengths

- Fred Hutchinson Cancer Center is well-equipped for such research.
- Washington State University and the BLACK HILLS CENTER FOR AMERICAN INDIAN HEALTH in South Dakota are adequately prepared.
- LOS from distinguished AIANs who will join the CAB.

Weaknesses

- No LOS or quote from Moby, and limited info provided on them. Mitigated by the fact that they have worked with the PI on similar projects previously.

Study Timeline:

Strengths

- Generally acceptable.

Weaknesses

- Updating the app in 9 months seems ambitious, particularly with limited info about Moby and no LOS from them.

Protections for Human Subjects:

Acceptable Risks and/or Adequate Protections.

- Will be reviewed by AIANs and Tribal representatives.

Data and Safety Monitoring Plan (Applicable for Clinical Trials Only):

Acceptable.

- Acceptable.

Inclusion Plans:

- Sex/Gender: Distribution justified scientifically.
- Race/Ethnicity: Distribution justified scientifically.
- For NIH-Defined Phase III trials, Plans for valid design and analysis: Scientifically acceptable.
- Inclusion/Exclusion Based on Age: Distribution justified scientifically.
- AIAN adults, quota to get 40% men, will assess sex/gender differences. Expect 15% Hispanic but will not be assessing by ethnicity.

Vertebrate Animals:

Not Applicable (No Vertebrate Animals)

Biohazards:

Not Applicable (No Biohazards)

Resubmission:

- Appropriately responsive to prior review.

Resource Sharing Plans:

Acceptable

- Project includes dissemination planning.

Budget and Period of Support:

[redacted]

[redacted] [redacted]

[redacted] [redacted].

CRITIQUE 3

Significance: 1
Investigator(s): 2
Innovation: 1
Approach: 3
Environment: 1

Overall Impact: This is a resubmission of an R01 application by an experienced NIH investigator. AI/ANs smoke cigarettes at double the rate of other ethnic groups and have 6x higher rates of smoking related cancers. Yet smoking cessation interventions have rarely been tested in this group. The investigators propose to test a digital smartphone app, called iCanQuit, to help AIANs stop smoking. Eligible AIANs will be randomized to either a modestly adapted iCanQuit group or to QuitGuide (total sample=786). Primary outcome will be a biochemically verified self-reported 30-day point prevalence abstinence (PPA) measure. Mediator and moderator analyses will be conducted, which will help demonstrate how the intervention is effective. A final aim centers on doing a qualitative assessment of users experience with the app, and to plan for dissemination. The proposed study has many strengths, including high significance, a strong and experienced study team, great innovation and approach. Preliminary data support the efficacy of the iCanQuit app in AIAN peoples. Preliminary work has been done to identify five key areas of adaptation of iCanQuit – this is their plan for refinement of iCANQuit for AIANs. A very strong and detailed plan is provided for setting up and activities of a CAB. Well thought out recruitment strategy based on experience. The researchers propose qualitative interviews with (1) a subsample of iCanQuit participants to provide themed testimonials of their experience with iCanQuit and (2) AIAN members from a study Community Advisory Board (CAB) will help disseminate iCanQuit to AIAN adults nationwide. The proposed study has a few minor weaknesses, which are outweighed by the many strengths of the study. Study team could use an implementation scientist considering some of the issues of acceptability and accessibility being studied. There appears to be a lack of a heavily experienced qualitative researcher on the team, though clearly some have experience in these methods. The iCanQuit intervention seems strictly adhered to (with five changes), which feels limiting. It's unclear how the app will allow for individual tailoring. Can an individual intervention like this work in collectively oriented cultural setting? – seems like it might be more effective in very acculturated individuals. No ethnic identify measure is provided, which might help understand effects within subgroups. The study does not seem to be operating from a clear theoretical or conceptual behavior change framework other than Acceptance and Commitment Therapy (ACT) – what is the theoretical basis for iCanQuit? Seems a need for implementation science if a goal is to improve accessibility. Measures of engagement section seems to be a step in this direction.

1. Significance:

Strengths

- American Indians and Alaska Natives (AIANs) have maintained the highest rates of commercial cigarette smoking of any racial/ethnic group in the US. Currently, 27% of AIANs smoke cigarettes.
- AIAN lack efficacious smoking cessation interventions.
- Apps require no in-person delivery and no provider training, do not require integration into complex hospital systems, can be freely accessed on an app store, and are available at any time and any place.

- Apps have potentially high population-level reach to AIANs given that 68-78% of AIANs own smartphones.

Weaknesses

- None noted by reviewer.

2. Investigator(s):

Strengths

- Excellent team with expertise in clinical psychology, nutrition, clinical/intervention trials, health policy, medicine, statistics.
- Team has long-standing collaborations with tribal communities in developing and implementing culturally relevant research projects including smoking AIANs, cessation strategies for and smoking policy interventions as well as engaging with local tribal leaders and traditional healers, using their knowledge and beliefs about smoking in public places and ceremonies to effectively implement and promote smoke free policies.

Weaknesses

- There does not appear to be a co-investigator with named and heavy expertise in qualitative research methods (Nelson?)
- Health economist could be used if assessing the value of this apparently highly cost-effective intervention.

3. Innovation:

Strengths

- First full-scale RCT testing any digital intervention for helping AIANs stop smoking nationwide.
- Will explore strategies to disseminate app for AIAN populations.

Weaknesses

- None noted by reviewer.

4. Approach:

Strengths

- Preliminary data support the efficacy of the iCanQuit app in AIAN peoples.
- Preliminary work done to identify five key areas of adaptation of iCanQuit – this is their plan for refinement of iCANQuit for AIANs. Aim to ensure accessibility, not to boost cessation efficacy.
- Very strong and detailed plan for setting up and activities of a CAB.
- Well thought out recruitment strategy based on experience.
- Propose qualitative interviews with (1) a subsample of iCanQuit participants to thematize testimonials of their experience with iCanQuit and (2) AIAN members from our study Community Advisory Board (CAB) to help disseminate iCanQuit to AIAN adults nationwide.

Weaknesses

- No cost information is being collected – which seems a missed opportunity.

- Don't fully accept the argument for lack of strong cultural adaptation – particularly in terms of tailoring for individuals – intervention seems bound by the previous intervention from which it is drawn.
- Does not seem to be operating from a theoretical or conceptual behavior change framework other than Acceptance and Commitment Therapy (ACT) – what is the basis for iCanQuit?
- Seems a need for implementation science if a goal is to improve accessibility. Measures of engagement section seems to be a step in this direction, but are incomplete.
- Can an individual intervention like this work in collectively oriented cultural setting? Seems like it might be more effective in very acculturated individuals. Additional measures of ethnic identity could be useful.

5. Environment:

Strengths

- Great university setting and resources.
- Excellent and meaningful partnership with CBOs focusing on ANANs.

Weaknesses

- None noted by reviewer.

Study Timeline:

Strengths

- Present.

Weaknesses

- None noted by reviewer.

Protections for Human Subjects:

Acceptable Risks and/or Adequate Protections.

Data and Safety Monitoring Plan (Applicable for Clinical Trials Only):

Acceptable

Inclusion Plans:

- Sex/Gender: Distribution justified scientifically.
- Race/Ethnicity: Distribution justified scientifically.
- For NIH-Defined Phase III trials, Plans for valid design and analysis: Scientifically acceptable.
- Inclusion/Exclusion Based on Age: Distribution justified scientifically.

Vertebrate Animals:

Not Applicable (No Vertebrate Animals)

Biohazards:

Not Applicable (No Biohazards)

Resubmission:

- Highly responsive to previous review -- such as the addition of a dissemination plan.

Resource Sharing Plans:
Not Applicable (No Relevant Resources)

Budget and Period of Support:
Recommend as Requested.

THE FOLLOWING SECTIONS WERE PREPARED BY THE SCIENTIFIC REVIEW OFFICER TO SUMMARIZE THE OUTCOME OF DISCUSSIONS OF THE REVIEW COMMITTEE, OR REVIEWERS' WRITTEN CRITIQUES, ON THE FOLLOWING ISSUES:

PROTECTION OF HUMAN SUBJECTS: ACCEPTABLE

INCLUSION OF WOMEN PLAN: ACCEPTABLE

INCLUSION OF MINORITIES PLAN: ACCEPTABLE

INCLUSION ACROSS THE LIFESPAN: ACCEPTABLE

COMMITTEE BUDGET RECOMMENDATIONS: The budget was recommended as requested.

Footnotes for 1R01CA284687-01A1; PI Name: Bricker, Jonathan B

NIH has modified its policy regarding the receipt of resubmissions (amended applications).See Guide Notice NOT-OD-18-197 at https://grants.nih.gov/grants/guide/notice-files/NOT-OD-18-197.html. The impact/priority score is calculated after discussion of an application by averaging the overall scores (1-9) given by all voting reviewers on the committee and multiplying by 10. The criterion scores are submitted prior to the meeting by the individual reviewers assigned to an application, and are not discussed specifically at the review meeting or calculated into the overall impact score. Some applications also receive a percentile ranking. For details on the review process, see http://grants.nih.gov/grants/peer_review_process.htm#scoring.

MEETING ROSTER

Health Promotion in Communities Study Section
Healthcare Delivery and Methodologies Integrated Review Group
CENTER FOR SCIENTIFIC REVIEW
HPC
06/20/2023 - 06/21/2023

Notice of NIH Policy to All Applicants: Meeting rosters are provided for information purposes only. Applicant investigators and institutional officials must not communicate directly with study section members about an application before or after the review. Failure to observe this policy will create a serious breach of integrity in the peer review process, and may lead to actions outlined in NOT-OD-22-044 at https://grants.nih.gov/grants/guide/notice-files/NOT-OD-22-044.html, including removal of the application from immediate review.

CHAIRPERSON(S)

NICCOLAI, LINDA M, PHD
PROFESSOR AND ASSOCIATE DEAN
DEPARTMENT OF EPIDEMIOLOGY OF MICROBIAL DISEASES
YALE SCHOOL OF PUBLIC HEALTH
YALE UNIVERSITY
NEW HAVEN, CT 06520

MEMBERS

BERG, CARLA J, PHD
PROFESSOR
DEPARTMENT OF PREVENTION AND COMMUNITY HEALTH
GEORGE WASHINGTON UNIVERSITY
WASHINGTON, DC 20052

BOUSTANI, MAYA M, PHD *
ASSOCIATE PROFESSOR
DEPARTMENT OF PSYCHOLOGY
SCHOOL OF BEHAVIORAL HEALTH
LOMA LINDA UNIVERSITY
LOMA LINDA, CA 92350

BURKE, MEGHAN, PHD *
PROFESSOR
DEPARTMENT OF SPECIAL EDUCATION
UNIVERSITY OF ILLINOIS URBANA-CHAMPAIGN
CHAMPAIGN, IL 61820

CALO, WILLIAM ALEXIS, JD, MPH, PHD *
ASSISTANT PROFESSOR
DIVISION OF HEALTH SERVICES
AND BEHAVIORAL RESEARCH
DEPARTMENT OF PUBLIC HEALTH SCIENCES
PENN STATE UNIVERSITY HERSHEY MEDICAL CENTER
HERSHEY, PA 17033

CRESPO, NOE C, PHD *
PROFESSOR
DIVISION OF HEALTH PROMOTION
AND BEHAVIORAL SCIENCE
SCHOOL OF PUBLIC HEALTH
SAN DIEGO STATE UNIVERSITY
SAN DIEGO, CA 92182

GITTELSOHN, JOEL, PHD
PROFESSOR
DEPARTMENT OF INTERNATIONAL HEALTH
CENTER FOR HUMAN NUTRITION
BLOOMBERG SCHOOL OF PUBLIC HEALTH
JOHNS HOPKINS UNIVERSITY
BALTIMORE, MD 21205

GRINER, STACEY B, MPH, PHD *
ASSISTANT PROFESSOR
SCHOOL OF PUBLIC HEALTH
UNIVERSITY OF NORTH TEXAS HEALTH SCIENCE CENTER
FORT WORTH, TX 76107

HECKMAN, CAROLYN J, PHD
PROFESSOR OF MEDICINE
CO-LEADER, CANCER PREVENTION AND CONTROL
PROGRAM
RUTGERS CANCER INSTITUTE OF NEW JERSEY
RUTGERS UNIVERSITY
NEW BRUNSWICK, NJ 08901

ISLAM, NADIA S, PHD
ASSOCIATE PROFESSOR
DEPARTMENT OF POPULATION HEALTH
NEW YORK UNIVERSITY SCHOOL OF MEDICINE
NEW YORK, NY 10016

KARPYN, ALLISON E, PHD *
ASSOCIATE PROFESSOR
CENTER FOR RESEARCH IN EDUCATION
AND SOCIAL POLICY
SCHOOL OF EDUCATION
UNIVERSITY OF DELAWARE
NEWARK, DE 19716

KARR, CATHERINE J, MD, PHD
PROFESSOR
DEPARTMENTS OF PEDIATRICS AND
ENVIRONMENTAL AND OCCUPATIONAL HEALTH SCIENCES
UNIVERSITY OF WASHINGTON
SEATTLE, WA 98105

KIVINIEMI, MARC T, PHD *
PROFESSOR
DEPT OF HEALTH, BEHAVIOR, AND SOCIETY
UNIVERSITY OF KENTUCKY
LEXINGTON, KY 40536

LECHUGA, JULIA, PHD
ASSOCIATE PROFESSOR
DEPARTMENT OF PUBLIC HEALTH SCIENCES
THE UNIVERSITY OF TEXAS AT EL PASO
EL PASO, TX 79902

LEE, SUNMIN, MPH, SCD
PROFESSOR
DEPARTMENT OF MEDICINE
SCHOOL OF MEDICINE
UNIVERSITY OF CALIFORNIA, IRVINE
IRVINE, CA 92697

LYONS, ELIZABETH J, MPH, PHD
ASSOCIATE PROFESSOR
DEPARTMENT OF NUTRITION AND METABOLISM
THE UNIVERSITY OF TEXAS MEDICAL BRANCH
GALVESTON, TX 77550

MAIR, CHRISTINA FURBER, PHD
ASSOCIATE PROFESSOR
DEPARTMENT OF BEHAVIORAL AND
COMMUNITY HEALTH SCIENCES
SCHOOL OF PUBLIC HEALTH
UNIVERSITY OF PITTSBURGH
PITTSBURGH, PA 15261

NELSON, TOBEN FREDERICK, SCD
PROFESSOR
DIVISION OF EPIDEMIOLOGY
AND COMMUNITY HEALTH
SCHOOL OF PUBLIC HEALTH
UNIVERSITY OF MINNESOTA
MINNEAPOLIS, MN 55454

PATEL, MINAL R, PHD
ASSOCIATE PROFESSOR
DEPARTMENT OF HEALTH BEHAVIOR AND HEALTH
EDUCATION
SCHOOL OF PUBLIC HEALTH
UNIVERSITY OF MICHIGAN
ANN ARBOR, MI 48109

RALSTON, PENNY ANN, PHD
PROFESSOR
DEPARTMENT OF HUMAN DEVELOPMENT AND FAMILY
SCIENCE
DEAN EMERITUS, COLLEGE OF HLTH AND HUMAN
SCIENCES
FLORIDA STATE UNIVERSITY
TALLAHASSEE, FL 32306

ROSAS, LISA GOLDMAN, PHD
ASSISTANT PROFESSOR
DEPARTMENT OF EPIDEMIOLOGY AND POPULATION
HEALTH
DEPARTMENT OF MEDICINE
STANFORD UNIVERSITY SCHOOL OF MEDICINE
PALO ALTO, CA 94305

SERVIN, ARGENTINA ELISA NOELLE, MD MPH *
ASSISTANT ADJUNCT PROFESSOR
DIVISION OF INFECTIOUS DISEASES AND
GLOBAL PUBLIC HEALTH
SCHOOL OF MEDICINE
UNIVERSITY OF CALIFORNIA SAN DIEGO
LA JOLLA, CA 92093

WANG, RUI, PHD
ASSOCIATE PROFESSOR
DEPARTMENT OF POPULATION MEDICINE
HARVARD PILGRIM HEALTH CARE INSTITUTE
HARVARD MEDICAL SCHOOL
BOSTON, MA 02215

WINTERS-STONE, KERRI M, PHD
PROFESSOR
DIVISION OF ONCOLOGICAL SCIENCES
KNIGHT CANCER INSTITUTE
OREGON HEALTH AND SCIENCE UNIVERSITY
PORTLAND, OR 97239

WIPPOLD, GUILLERMO MANUEL, PHD *
ASSISTANT PROFESSOR
DEPARTMENT OF PSYCHOLOGY
UNIVERSITY OF SOUTH CAROLINA
COLUMBIA, SC 29208

SCIENTIFIC REVIEW OFFICER

DAGADU, HELENA ERYAM, MPH, PHD
SCIENTIFIC REVIEW OFFICER
CENTER FOR SCIENTIFIC REVIEW
NATIONAL INSTITUTES OF HEALTH
BETHESDA, MD 20892

EXTRAMURAL SUPPORT ASSISTANT

GUERRERO, KATHERINE
EXTRAMURAL SUPPORT ASSISTANT
THE CENTER FOR SCIENTIFIC REVIEW
THE NATIONAL INSTITUTES OF HEALTH
BETHESDA, MD 20892

* Temporary Member. For grant applications, temporary members may participate in the entire meeting or may review only selected applications as needed.

Consultants are required to absent themselves from the room during the review of any application if their presence would constitute or appear to constitute a conflict of interest.
